# Knee loading reduces MMP13 activity in the mouse cartilage

**DOI:** 10.1186/1471-2474-14-312

**Published:** 2013-11-01

**Authors:** Kazunori Hamamura, Ping Zhang, Liming Zhao, Joon W Shim, Andy Chen, Todd R Dodge, Qiaoqiao Wan, Han Shih, Sungsoo Na, Chien-Chi Lin, Hui Bin Sun, Hiroki Yokota

**Affiliations:** 1 Department of Biomedical Engineering, Indiana University Purdue University Indianapolis, SL220C, 723 West Michigan Street, Indianapolis, IN 46202, USA; 2 School of Basic Medical Sciences, Tianjin Medical University, Tanjin 300070, People’s Republic of China; 3 Department of Orthopaedic Surgery, Albert Einstein College of Medicine, Bronx, NY 10461, USA; 4 Department of Anatomy and Cell Biology, Indiana University School of Medicine, Indianapolis, IN 46202, USA

**Keywords:** Knee loading, Cartilage, Chondrocyte, Osteoarthritis, MMP13, Rac1

## Abstract

**Background:**

Moderate loads with knee loading enhance bone formation, but its effects on the maintenance of the knee are not well understood. In this study, we examined the effects of knee loading on the activity of matrix metalloproteinase13 (MMP13) and evaluated the role of p38 MAPK and Rac1 GTPase in the regulation of MMP13.

**Methods:**

Knee loading (0.5–3 N for 5 min) was applied to the right knee of surgically-induced osteoarthritis (OA) mice as well as normal (non-OA) mice, and MMP13 activity in the femoral cartilage was examined. The sham-loaded knee was used as a non-loading control. We also employed primary non-OA and OA human chondrocytes as well as C28/I2 chondrocyte cells, and examined MMP13 activity and molecular signaling in response to shear at 2–20 dyn/cm^2^.

**Results:**

Daily knee loading at 1 N for 2 weeks suppressed cartilage destruction in the knee of OA mice. Induction of OA elevated MMP13 activity and knee loading at 1 N suppressed this elevation. MMP13 activity was also increased in primary OA chondrocytes, and this increase was attenuated by applying shear at 10 dyn/cm^2^. Load-driven reduction in MMP13 was associated with a decrease in the phosphorylation level of p38 MAPK (p-p38) and NFκB (p-NFκB). Molecular imaging using a fluorescence resonance energy transfer (FRET) technique showed that Rac1 activity was reduced by shear at 10 dyn/cm^2^ and elevated by it at 20 dyn/cm^2^. Silencing Rac1 GTPase significantly reduced MMP13 expression and p-p38 but not p-NFκB. Transfection of a constitutively active Rac1 GTPase mutant increased MMP13 activity, while a dominant negative mutant decreased it.

**Conclusions:**

Knee loading reduces MMP13 activity at least in part through Rac1-mediated p38 MAPK signaling. This study suggests the possibility of knee loading as a therapy not only for strengthening bone but also preventing tissue degradation of the femoral cartilage.

## Background

Moderate mechanical loading applied laterally to the knee has been shown to enhance bone formation [1] and attenuate pain perception-linked signaling [2], but the effect of lateral loads to the knee on the maintenance of cartilage tissues remains undetermined [3]. The major cellular subpopulation in cartilage is constituted by chondrocytes, which are critical due in part to their role in biosynthesis of extracellular matrix as well as secretion of matrix metalloproteinases (MMPs) such as MMP1, MMP3, and MMP13. Excess loads are considered to induce degenerative activities of collagenases and gelatinases in chondrocytes, while unloading due to immobilization also presents detrimental outcomes [4,5]. While anabolic responses to bone by various mechanical loading modalities have been demonstrated, little is known about the effects of mechanical loading to the knee on the regulation of MMPs in chondrocytes of the femoral cartilage in the knee.

Knee loading applies dynamic lateral loads to the knee joint and stimulates bone formation not only in the distal femur, but along the entire length of the femur [6,7]. The loading force required to stimulate bone formation due to knee loading is lower than that of other mechanical loading regimens, and strains in areas of bone formation are also reduced. This characteristic makes knee loading an attractive potential treatment in accelerating fracture repair [1]. Fractures of the femoral neck, which are a serious public health concern, experience faster healing times as a result of dynamic knee loading [8]. To explain the observed increases in bone formation due to knee loading, studies have focused on biophysical and molecular mechanisms that occur during and following a loading treatment [9-11]. Dynamic deformations of the epiphysis cause alterations in fluid pressure in the intramedullary cavity, driving oscillatory fluid flow and molecular transport in the lacunocanalicular network in the bone matrix and in the medullary cavity [10]. Fluid flow may cause shear stress to osteocytes, leading to osteoblast differentiation and the initiation of bone formation [12]. Although loads are directly applied to the knee, loading effects on maintenance of cartilage tissue and chondrocytes have not been examined.

Herein we addressed a question: Can knee loading, which gently applies lateral loads to the knee, reduce degenerative activity of MMP13 in non-OA and OA cartilage in the femur? If yes, what signaling pathway mediates load-driven suppression of MMP13 activity in chondrocytes? We hypothesized that the responses to knee loading are dependent on loading intensity. Mild and moderate loads (0.5–1 N) may reduce MMP13 activity, while strong loads (3 N) may elevate it. We also hypothesized that signaling pathways linked to inflammation, and cellular proliferation and differentiation are possibly involved in the regulation of MMP13 through p38 MAPK, NFκB, or small GTPases such as Rac1. The p38 MAPK pathway is responsive to mechanical stimulation and involved in the expression of MMP13 [13], while the NFκB pathway is known to be involved in inflammation and tissue degradation [14]. Furthermore, Rac1 GTPase is known to regulate cytoskeletal shape and activate p38 MAPK [15,16].

In testing the above hypothesis, we applied knee loading to the right knee of non-OA and OA mice and provided fluid flow shear to primary human chondrocytes (h-nonOA control, and h-OA) together with a C28/I2 human chondrocyte cell line. To evaluate potential dependence on loading magnitude, knee loading was applied at three levels of 0.5 N (mild), 1 N (moderate), and 3 N (strong), and fluid flow was given to induce shear intensity of 2 and 5 dyn/cm^2^ (mild), 10 dyn/cm^2^ (moderate), and 20 dyn/cm^2^ (strong). Activities of collagenases, gelatinases, and MMP13 were determined using fluorescent substrates specific to their activities, and the MMP13 mRNA level was determined using quantitative real-time PCR. Western blot analysis was performed to determine the phosphorylation levels of p38 MAPK and NFκB, and activity of Rac1 GTPase was monitored using a fluorescence resonance energy transfer (FRET) technique with a biosensor specific to Rac1 GTPase. To evaluate the role of Rac1 GTPase in the regulation of MMP13, silencing of Rac1 was conducted using siRNA specific to Rac1. Furthermore, two Rac1 GTPase mutants (Rac1-L61 and Rac1-N17) were transfected to C28/I2 chondrocyte cells and the effects of dominant negative and constitutively active forms of Rac1 GTPase were evaluated on MMP13 activity.

## Methods

### Animals and knee loading

**Figure 1:**
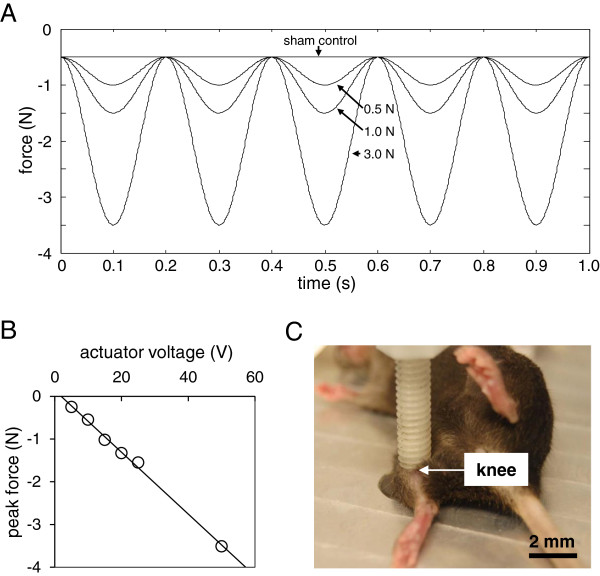
**Knee loading and histological observation of the mouse knee. (A)** Force profiles for knee loading. A 5 Hz sinusoidal load was applied to the right knee at a magnitude of 0.5 N, 1.0 N, or 3.0 N (peak-to-peak). Sham-loaded controls received a static load of 0.5 N. **(B)** Force-voltage relationship. To verify peak forces applied to the knee, the loading device was calibrated using a load cell and a linear relationship was defined relating actuator voltage and peak compressive force. **(C)** Application of knee loading. A custom-made piezoelectric loader was utilized to load the mouse knee.

Animal procedures were approved by the Indiana University IACUC. Fifty-seven C57/BL/6 female mice (~12 weeks, Harlan Laboratories) were used. OA in the right knee was surgically induced by transecting the medial collateral ligament and removing the medial meniscus [17]. Using the procedure previously described [6-8], knee loading was applied to the right limb using a piezoelectric loader (Figures 1A-C). The loading device was calibrated using a load cell (Model LLB130, FUTEK Advanced Sensor Technology, Irvine, CA) to determine peak compressive force for increasing actuator voltage. Pre-load was established at 0.5 N, and peak forces during three trials at each of six input voltages were averaged using manufacturer-provided software to establish a linear equation relating actuator voltage and peak compressive force. The mouse was anesthetized using 2% isoflurane and lateral dynamic loads to the knee were applied for 5 min. Loads were sinusoidal at 0.5, 1, or 3 N (peak-to-peak) and the frequency was 5 Hz. The anesthetized mice placed on the loading device were used as the sham-loaded control. The femoral cartilage was harvested 1 h after the loading bout.

### Histology

Knee samples were decalcified in 14% EDTA for 2 weeks, embedded in paraffin, sectioned at 4 μm thickness, and placed on glass slides. For staining, slides were deparaffinized and hydrated with distilled water. Slides were first stained with Weigert’s iron hematoxyline solution for 5 min and washed in distilled water. Next, slides were differentiated in 1% acid-alcohol for 2 sec, rinsed in distilled water and stained with 0.02% fast green solution for 1 min. They were then rinsed in 1% acetic acid for 30 sec, stained in 1% Safranin O solution for 30 min and dehydrated and treated with graded ethyl alcohol and xylene.

### Historogical grading for osteoarthritic changing

Tissue sections were stained with Safranin O and graded by two blinded observers based on the scoring system developed by Chambers et al. [18]. In brief, section was assigned a grade as follows: 0 = normal cartilage without any superficial zone fibrillation or clefting; 1 = mild superficial fibrillation without loss of cartilage; 2 = loss of surface lamina and fibrillations extending down to the calcified cartilage; 3 = mild loss of < 25% articular calcified length; 4 = moderate loss of 25-50% articular calcified cartilage length; 5 = loss of 25-50% articular carcified cartilage length; 6 = severe loss of > 75% articular calcified cartilage length. This method focuses on the structural changes and cartilage erosion degree. We use Image J to measure the articular cartilage length and evaluate the total surface length, then obtained the ratio and grades. Grades for all slides from each sample were averaged.

### Bone mineral density (BMD) and bone mineral content (BMC) in the knee

Among 3 groups of animals (age-matched control, OA control, and OA with knee loading; 7 mice per group), surgical induction of OA was conducted to the right knee of the mice in OA control and OA with knee loading. On day 8 after induction of OA, knee loading (1 N at 5 Hz for 5 min) was applied daily for 2 weeks to the mice in the group of OA with knee loading. Animals were sacrificed, and their right knee joints were harvested. Using peripheral dual energy X-ray absorptiometry (DXA; PIXImus II, Lunar Corp., Madison, WI, USA) and its software (version 1.47) [8], BMD (g/cm^2^) and BMC (g) were determined focusing on 3 regions of interest, including the entire knee (5 mm in length including the distal femur and the proximal tibia), the distal femur (2.5 mm in length from the distal edge of the femur), and the proximal tibia (2.5 mm in length from the proximal edge of the tibia).

### Primary human chondrocytes from normal and osteoarthritic tissues

Human primary chondrocytes (h-OA) from OA patient were harvested, and normal (h-nonOA) primary chondrocytes were purchased (PC136121A1-C, Asterand). We received necessary consent from the patients involved in the study. The OA cartilage was dissected into small pieces and digested in pronase (1 mg/ml, Roche) for 30 min followed by digestion in collagenase P (1 mg/ml, Roche). Cells were maintained in DMEM/F-12 medium containing 10% FBS and antibiotics (Invitrogen).

### Application of fluid flow shear to C28/I2 Chondrocyte cells

Human C28/I2 chondrocyte cells [13] were cultured on a glass slide coated with 1% type 1 collagen (BD Biosciences) in DMEM containing 10% FBS. Prior to mechanical stimulation, cells were grown in the medium containing 0.5% FBS for 24 h. Using the procedure previously described [19], fluid flow shear at 5 dyn/cm^2^ was applied to h-OA1 chondrocyte cells with a streamer fluid flow device (Flexcell International).

### Assays for activities of collagenases, gelatinases, and MMP13

To assay activities of collagenases and gelatinases, an EnzChek collagenase/gelatinase assay kit (E12055, Molecular Probes) was employed [20]. MMP13 sensitive fluorogenic peptide probe (Gly-Pro-Leu-Gly-Val-Arg-Gly-Cys-Gly-Gly) was prepared using a microwave peptide synthesizer (CEM Discover SPS) [21]. The isolated proteins were incubated with the fluorescent substrates for 2 h. Fluorescent intensity, a measure of enzymatic activity, was determined at excitation/emission ratio of 495/515 nm (collagenase and gelatinase) and 568/591 nm (MMP13).

### Western blot analysis

Protein samples were isolated from cartilage tissues and C28/I2 chondrocyte cells. Cartilage tissues were dissociated with a mortar and pestle in a RIPA lysis buffer containing inhibitors for proteases and phosphatases (Calbiochem). Isolated proteins were fractionated using SDS gels and electro-transferred to membranes. Immunoblots were performed using primary antibodies specific to p38 MAPK, p-p38 MAPK, NFκB p65 (Rel A), p-NFκB (Cell Signaling), and β-actin (Sigma). After incubation with secondary IgG antibodies conjugated with horseradish peroxidase, signals were detected with enhanced chemiluminescence.

### Real-time PCR

Total RNA was isolated from cartilage tissues and C28/I2 chondrocyte cells using RNeasy Plus Mini kits (Qiagen). Cartilage tissues were dissociated with a mortar and pestle in a guanidine thiocyanate lysis buffer consisting of 1% (v/v) 2-mercaptoethanol. The mRNA level of MMP13 was determined using quantitative PCR with a pair of primers (forward: 5′-GCA ACA AAG TAG ATG CTG TCT ATG AGA-3′; and reverse: 5′-ATG CGA TTA CTC CAG ATA CTG TAT TCA A-3′). Total RNA was extracted using an RNeasy Plus mini kit (Qiagen). Reverse transcription was performed, and real-time PCR was carried out using SYBR green PCR kits (Applied Biosystems). The mRNA level of GAPDH was used as control with a pair of primers (forward: 5′-TGC ACC ACC AAC TGC TTA G-3′; and reverse: 5′-GGA TGC AGA GAA GAT GTT C-3′).

### Transfection of Rac1 GTPase siRNA as well as dominant negative and constitutively active Rac1 GTPase mutants

To evaluate the role of Rac1 GTPase in regulation of MMP13, siRNA specific to Rac1 GTPase (5′-AGG GUC UAG CCA UGG CUA AGG AGA U-3′; Invitrogen) was transiently transfected to C28/I2 chondrocyte cells. Cells were transfected in Opti-MEM I medium with Lipofectamine RNAiMAX, and non-specific siRNA (NC siRNA, Invitrogen) was used as a control. To further evaluate the role of Rac1 GTPase, two Rac1 mutants (Rac1-L61 and Rac1-N17) were transfected to C18/I2 chondrocyte cells. Rac1-L61 is a constitutively active (CA) Rac1 GTPase, while Rac1-N17 a dominant negative (DN) Rac1 GTPase [22]. These mutants were transfected using a Neon transfection system (Invitrogen), and the transfected cells were incubated in DMEM containing 0.5% FBS for 24–36 h.

### Fluorescence resonance energy transfer (FRET)

To evaluate Rac1 activity in C28/I2 chondrocyte cells in response to fluid flow shear [23,24], FRET imaging was conducted using a cyan fluorescent protein (CFP)-yellow fluorescent protein (YFP) Rac1 biosensor [25]. Cells were grown in μ-slide cell culture chambers (Ibidi) and exposed to unidirectional shear stress of 2–20 dyn/cm^2^. Time-lapse fluorescent images were acquired at an interval of 2 min using the following filter sets: CFP excitation at 438 ± 24 nm (center wavelength ± bandwidth); CFP emission at 483 ± 32 nm; and YFP emission at 542 ± 27 nm. The level of Rac1 activity for individual cells was determined by computing an emission ratio of YFP/CFP. The emission ratio was normalized by a basal level of Rac1 activity, and the high emission ratio corresponded to activation of Rac1 GTPase.

### Statistical analysis

The mean and standard deviation or standard error of mean (histology score) were calculated and parametric statistical tests were conducted. Student’s t-test was conducted for a two-group comparison, and one-way ANOVA followed by a post-hoc test using Fisher’s protected least significant difference was employed for more than two-group comparison. Statistical significance was assumed at *p* < 0.05, and the asterisks (* and **) denote *p* < 0.05; and *p* < 0.01, respectively.

## Results

The animals tolerated the procedure of OA surgery and knee loading. No infections were detected, and we did not observe any abnormal behavior, weight loss, or diminished food intake.

### Suppression of cartilage destruction by 2-week knee loading

**Figure 2:**
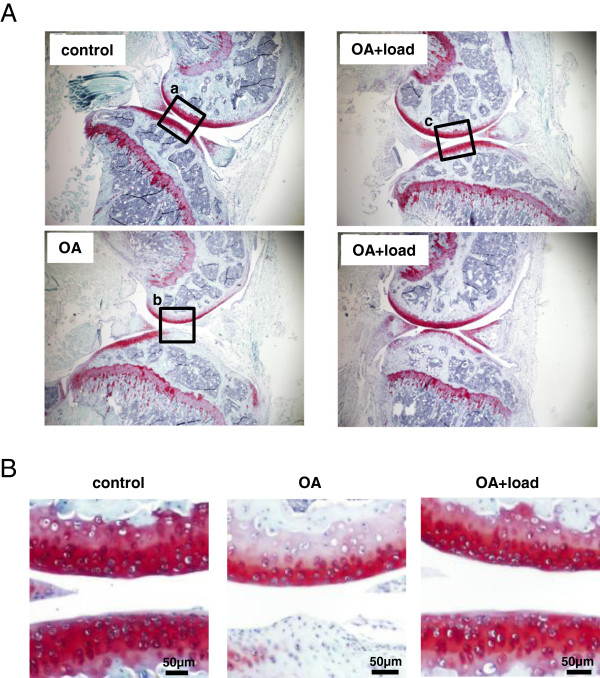
**Histological sections with Safranin O staining. (A)** Articular cartilage of the knee joint for the age-matched control, OA control without knee loading, and OA with knee loading. **(B)** Magnification of the squared regions. The OA control sample without knee loading presented a significant lack of staining, while the OA sample with knee loading restored Safranin O staining.

**Figure 3:**
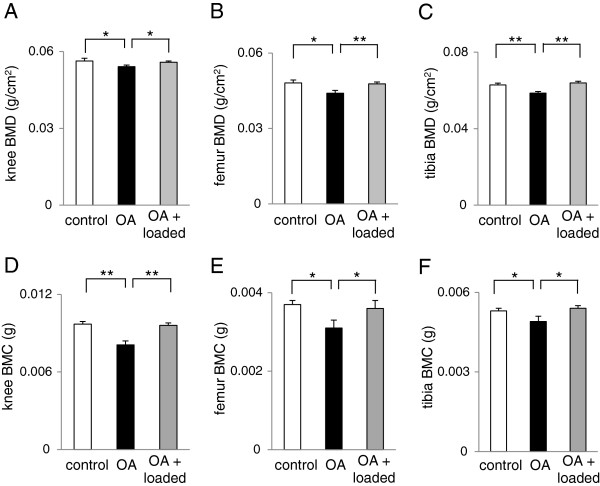
**BMD and BMC of the knee, distal femur, and proximal tibia in the three groups of mice (control, OA, and OA with knee loading). (A)** BMD of the entire knee. **(B)** BMD of the distal femur. **(C)** BMD of the proximal tibia. **(D)** BMC of the entire knee. **(E)** BMC of the distal femur. **(F)** BMC of the proximal tibia. n = 6 per group **(A-F)**.

Three weeks after induction of OA, the Safranin-O stained histological section of the right knee showed destruction of articular cartilage in the distal femur and proximal tibia (Figure 2A-B). When knee loading at 1 N was applied for 2 weeks 8 days after induction of OA, however, loss of surface staining was no longer observed (Figure 2B). In response to induction of OA, the histological score for the samples with knee loading was lowered compared to those without knee loading (age-matched control 4.29 ± 0.18; OA 5.3 ± 0.15; and OA + loaded 4.56 ± 0.18; N = 7). Furthermore, compared to the age-matched control, the OA control showed significant reduction in BMD and BMC in the entire knee, as well as in the distal femur and proximal tibia (Figure 3). Consistent with the histological observation of cartilage surfaces in the distal femur and proximal tibia, 2-week application of daily knee loading completely suppressed OA-induced reduction in BMD and BMC (Figure 3).

### Reduction of activities of collagenases, gelatinases, and MMP13 in non-OA mice by knee loading

**Figure 4:**
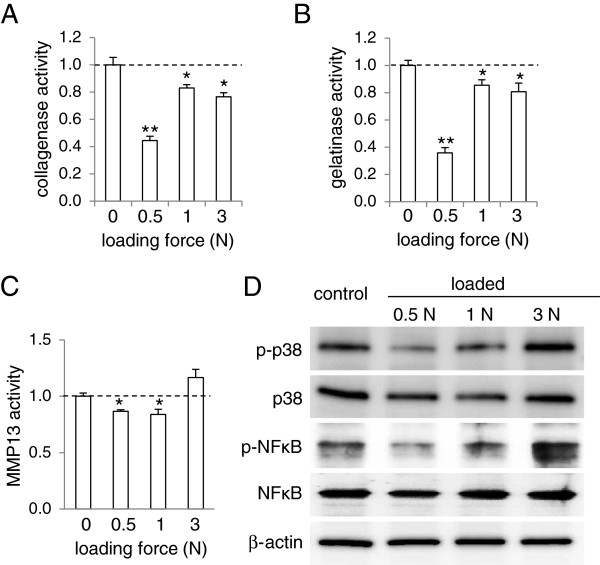
**Load-driven reduction in the activity levels of collagenases, gelatinases, and MMP13, as well as the phosphorylation levels of p38 MAPK and NFκB in normal mice. (A)** Reduction of collagenase activity in response to knee loading at 0.5, 1, and 3 N. The dotted line indicates the activity level of the sham loaded control without application of knee loading. **(B)** Reduction in gelatinase activity in response to knee loading at 0.5, 1, and 3 N. **(C)** Reduction in MMP13 activity in response to knee loading at 0.5 and 1 N. **(D)** Reduced phosphorylation levels of p38 MAPK (p-p38) and NFκB (p- NFκB) in response to knee loading at 0.5 and 1 N. n = 3 per group **(A-D)**.

To evaluate the effects of knee loading at the molecular level, we first examined activities of collagenases, gelatinases, and MMP13 in the femoral cartilage in the non-OA mice (Figure 4A-C). In response to loads at 0.5, 1, and 3 N, activities of collagenases and gelatinases were significantly reduced (Figure 4A-B). Furthermore, MMP13 activity was decreased by 13% and 16% in response to loads at 0.5 N and 1 N, respectively (Figure 4C). At the higher loads of 3 N, however, load-driven reduction of MMP13 was not observed (Figure 4C).

### Load-driven reduction of p-p38 and p-NFκB in non-OA mice

Loads at 0.5 and 1 N decreased the phosphorylated levels of p38 and NFκB in a load intensity-dependent manner (Figure 4D). The reduction of their phosphorylation levels was larger at 0.5 N than at 1 N. Loads at 3 N, however, did not lower the levels of p-p38, and they increased the level of p-NFκB.

### Elevation of MMP13 activity, p-p38, and p-NFκB in OA mice

**Figure 5:**
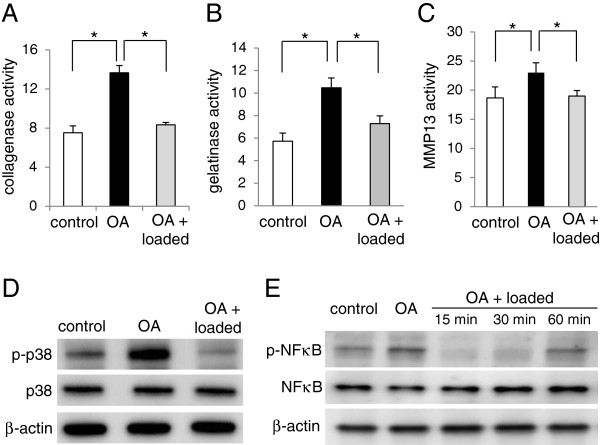
**Effects of knee loading (1 N) to OA mice on the activity levels of collagenases, gelatinases, and MMP13, as well as the phosphorylation levels of p38 MAPK and NFκB. (A)** Collagenase activity in the control, OA, and loaded OA cartilages. **(B)** Gelatinase activity in the control, OA, and loaded OA cartilages. **(C)** MMP13 activity in the control, OA, and loaded OA cartilages. **(D)** Levels of p-p38 in the control, OA, and loaded OA cartilages (60 min after knee loading). n = 3 per group **(A-D)**. **(E)** Levels of p-NFκB in the control, OA, and loaded OA cartilages (15, 30, and 60 min after knee loading). n = 3 per control, OA, and OA + loaded (15, 30, and 60 min), respectively.

Eight days after transection of the medical collateral ligament and a removal of the medial meniscus, activities of collagenases, gelatinases, and MMP13 were elevated by surgical induction of OA (Figure 5A-C). MMP13 activity, for instance, was increased by 21% in the femoral cartilage of the OA mice.

### Load-driven suppression of MMP13 activity, p-p38 and p-NFκB in OA mice

When 1 N loads were applied to the knee, the OA-driven upregulation of activities of collagenases, gelatinases, and MMP13 was significantly suppressed (Figure 5A-C). Consistent with the observed activities of collagenases, gelatinases, and MMP13, the levels of p-p38 and p-NFκB were increased by induction of OA and significantly reduced by knee loading at 1 N (Figure 5D-E).

### Effects of fluid flow shear on MMP13 activity in primary human chondrocytes

**Figure 6:**
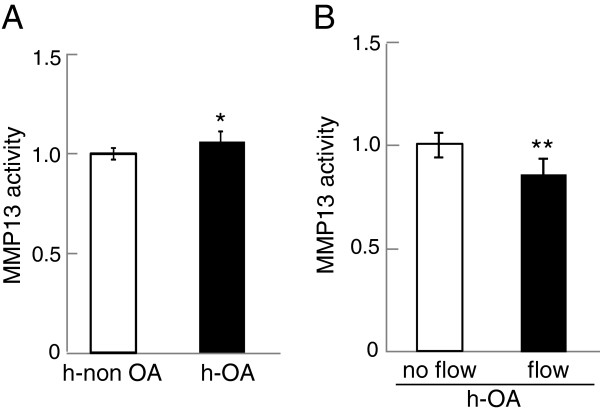
**MMP13 activity levels of primary chondrocyte cells isolated from normal cartilage tissue as well as OA cartilage tissues (h-OA). (A)** Comparison of MMP13 activity levels of normal and h-OA primary chondrocyte cells. **(B)** Reduction of MMP13 activity in h-OA primary chondrocyte cells in response to 1-hr fluid flow shear at 5 dyn/cm^2^. n = 3 per group **(A-B)**.

To evaluate MMP13 activity in primary human chondrocytes, we employed three samples each of non-OA cartilage (h-non OA) and OA cartilage (h-OA) samples. Compared to the non-OA chondrocyte cells, MMP13 activity was elevated in h-OA chondrocyte cells (*p* = 0.031) (Figure 6A). To examine the effects of mechanical stimulation to h-OA chondrocyte cells, we applied fluid flow shear at 5 dyn/cm^2^ for 1 h. In response to moderate fluid flow shear, MMP13 activity in h-OA chondrocyte cells was significantly reduced (*p* = 0.0093) (Figure 6B).

### FRET-based detection of flow-induced alteration in Rac1 activity

**Figure 7:**
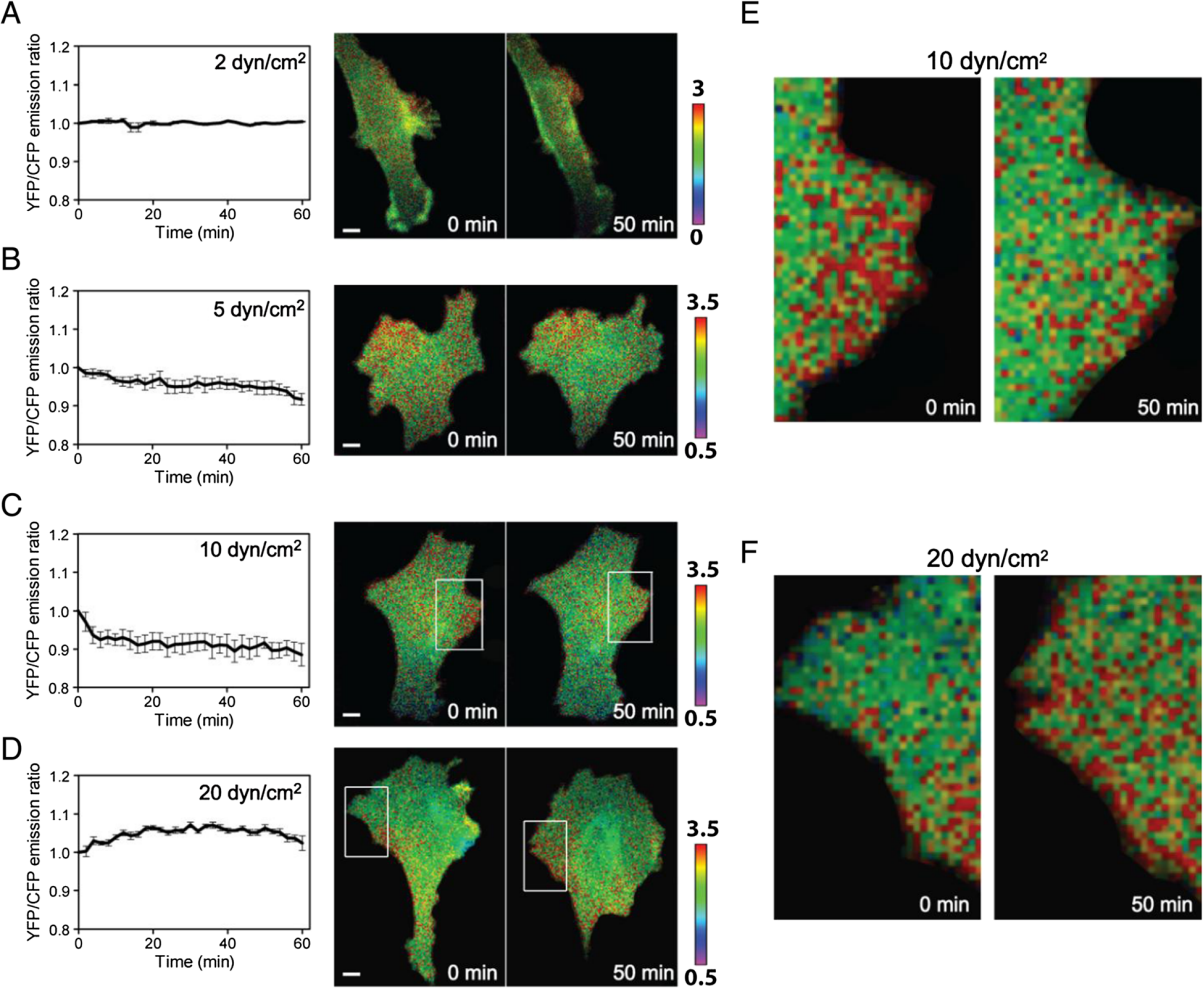
**FRET fluorescent images using the Rac1 GTPase biosensor in C28/I2 chondrocyte cells.** The red color indicates the activation level of Rac1. **(A-D)** Rac1 activity in response to fluid flow shear at 2, 5, 10, and 20 dyn/cm^2^ for 60 min. **(E)** Reduced Rac1 activity in response to fluid flow shear at 10 dyn/cm^2^ [zoom of the white boxes in **(C)**]. **(F)** Elevated Rac1 activity in response to fluid flow shear at 20 dyn/cm^2^ [zoom of the white boxes in **(D)**]. n = 7 to 17 **(A-F)**.

To evaluate the role of Rac1 GTPase in mechanical regulation of MMP13, Rac1 activity of C28/I2 chondrocyte cells was monitored using the Rac1 biosensor and FRET imaging. The cells were subjected to flow-induced shear stress at 2, 5, 10, or 20 dyn/cm^2^, and changes in the emission ratio of YFP/CFP of the Rac1 biosensor were determined. The result presented shear intensity-dependent regulation of Rac1 activity. At low fluid flow shear of 2 and 5 dyn/cm^2^, Rac1 activity was not significantly altered (Figure 7A-B). When shear intensity was raised to 10 dyn/cm^2^, however, it was continuously lowered in a 60-min observation period (Figure 7C). When shear intensity was further elevated to 20 dyn/cm^2^, Rac1 activity was significantly increased 20–50 min after the onset of fluid flow (Figure 7D). The spatial distribution of Rac1 activity was not uniform within individual cells. Depicted are the regions with high sensitivity (Figure 7E-F) for the responses to 10 and 20 dyn/cm^2^, respectively, where the red spots indicate activation of Rac1 GTPase.

### Downregulation of p-p38 and MMP13 expression by Rac1 siRNA

**Figure 8:**
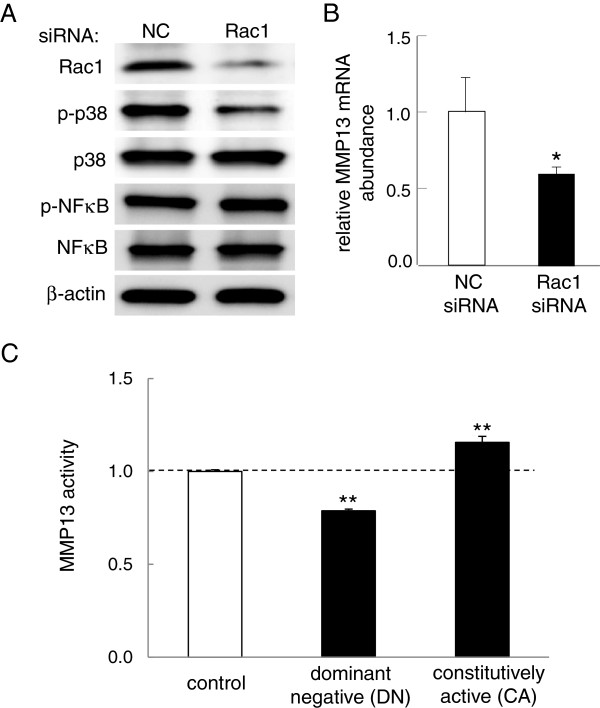
**Involvement of Rac1 GTPase in regulation of MMP13 in C28/I2 chondrocyte cells. (A)** Effects of silencing Rac1 on the phosphorylation level of p38 (p-p38) and NFκB (p-NFκB). The level of p-p38 was reduced, while the level of p-NFκB was unchanged. Note that NC is the non-specific control siRNA. **(B)** Decrease in the level of MMP13 mRNA in response to transfection of siRNA specific to Rac1 GTPase. **(C)** MMP13 activity in the cells transfected with the Rac1 dominant negative (DN) or constitutively active (CA) mutant. MMP13 activity was reduced by the DN mutant, which it was elevated by the CA mutant. n = 3 per group **(A-C)**.

Dependence of MMP13 activity and Rac1 GTPase activity on either loading intensity (in N) or fluid flow shear (in dyn/cm^2^) indicates a potential mechanism of Rac1 GTPase-mediated regulation of MMP13. We thus evaluated the effects of RNA interference with Rac1 siRNA on the expression of p-p38, p-NFκB, and MMP13. In C28/I2 chondrocyte cells, RNA interference with Rac1 siRNA significantly decreased the level of p-p38, but it did not alter the level of p-NFκB (Figure 8A). Compared to the control cells with NC siRNA, the cells transfected with Rac1 siRNA also presented significant reduction in the level of MMP13 mRNA (*p* = 0.045) (Figure 8B).

### Effects of two forms of Rac1 mutants on MMP13 activity

To further examine a potential linkage of Rac1 GTPase to regulation of MMP13, we transfected two types of Rac1 mutants to C28/I2 chondrocyte cells. In the cells transfected with dominant negative (DN) Rac1 mutants, MMP13 activity was significantly reduced by 21% (*p* = 0.001) (Figure 8C). In the cells transfected with constitutively active (CA) Rac1 mutants, MMP13 activity was elevated by 16% (*p* = 0.003) (Figure 8C).

## Discussion

We demonstrated that loading to the knee of non-OA and OA mice reduced activities of collagenases, gelatinases, and MMP13 in the femoral cartilage. We also showed that application of fluid shear at 10 dyn/cm^2^ to primary human OA chondrocytes downregulated their degenerative activities. Both mouse cartilage and human chondrocyte samples revealed that the reduction of these activities was associated with the decrease in the levels of p-p38 MAPK and p-NFκB. FRET imaging analysis using a Rac1-specific biosensor showed that Rac1 GTPase activity was lowered by fluid flow shear at 10 dyn/cm^2^. Furthermore, silencing Rac1 GTPase using RNA interference reduced MMP13 activity as well as the level of p-p38 but not p-NFκB. Also, transfection of the dominant negative Rac1 GTPase mutant reduced MMP13 activity, while that of the constitutively active Rac1 GTPase mutant elevated it. These results support the notion that moderate mechanical stimulation with knee loading to the femoral cartilage and fluid flow shear to chondrocytes attenuates MMP13 activity at least in part through Rac1 GTPase-mediated p38 MAPK signaling.

It is reported that knee loading with 0.5 N and 1 N loads at 1–20 Hz can induce anabolic responses throughout the femur when applied 3–5 min per day for 5 days [6,7]. In this study we employed the same loads of 0.5 N and 1 N as well as higher loads of 3 N and applied them at 5 Hz for 5 min. Although loads at 5 Hz were effective to attenuate MMP13 activity as well as activities of collagenases and gelatinases, loading efficacy may depend not only on loading magnitudes (in N) but also loading frequencies (in Hz) and duration (in min). We evaluated enzymatic activities 1 h after a single loading bout, but loading effects may differ depending on the number of loading bouts and time after loading bouts. Although oscillatory compressive stress at 0.2–0.5 atmospheric pressure (2–5 × 10^5^ dyn/cm^2^) was also able to reduce the levels of MMP13 mRNA and p-p38 in C28/I2 chondrocyte cells, cells were ~10^5^ times more sensitive to shear stress than compressive stress (data not shown). To conduct *in vitro* analysis of chondrocytes, we thus employed fluid flow shear at 2–20 dyn/cm^2^ that is considered to be induced in cartilaginous tissue by knee loading.

The FRET images using the Rac1 biosensor revealed that Rac1 activity was dependent on shear intensity and was significantly downregulated by fluid flow shear at 10 dyn/cm^2^. Rac1 is necessary for development and maintenance of cartilage [26], and its chondrocyte-specific deletion results in severe dwarfism in mice [27]. In response to fibronectin fragments, it is reported that Rac1 is required for the production of MMP13 in chondrocytes [28]. Using C28/I2 chondrocyte cells, we have shown that silencing Rac1 by siRNA reduces the activation of p38 MAPK, which is reported to be linked to the upregulation of MMP13 by inflammatory cytokines [13,29] and in osteosarcomas [30]. Rac1 and other members of the small Rho GTPase family such as RhoA and cdc42 are central regulators of cell motility and cytoskeleton [31,32]. We have previously observed a shear-intensity dependent regulation of RhoA in osteoblast cells [25], but we did not detect linkage of activity of RhoA GTPase to the regulation of MMP13 (data not shown). The role of GTPases in regulation of MMP13 could be dissimilar in different types of cells. MMP13 activity is reported to be regulated by NFκB as well as p38 MAPK signaling [14]. In this study, moderate knee loading as well as fluid flow shear reduced the level of both p-p38 and p-NFκB, but the activation of Rac1 GTPase was linked to p-p38 and not to p-NFκB. The previous reports on transcriptome-wide analysis and cell culture under compressive loads also suggest a pivotal role for the NFκB pathway in arthritis [33,34]. Further studies are necessary to determine the regulatory pathway of p-NFκB in response to knee loading.

Effects of knee loading may depend on the stage of OA development. In this study, we applied knee loading 1 week after surgical induction of moderate OA and evaluated MMP13 activity in the femoral cartilage. Transection of the medial collateral ligament induced misalignment of the joint rotation in the knee, and the removal of the medial meniscus generated abnormal friction on the surface of the femoral cartilage. Histological observation of the knee joint suggested that in 3 weeks the surgical induction of OA exhibited a clear defect of articular cartilage as well as a partial exposure of subchondral bone on the articular surface. In order to further evaluate the potential benefits of knee loading, it is imperative to examine its effects at different stages of OA development.

The current study focused on the effects of knee loading and fluid flow shear on the regulation of MMP13 in non-OA and OA cartilage tissues, as well as normal and OA chondrocyte cells. The causes and symptoms of OA differ among individual patients, and not only MMP13 but also other MMPs and ADAMTS (a disintegrin and metalloproteinase with thrombospondin motifs) are involved in the degradation of arthritic tissues. Furthermore, the regulation of MMPs in other joint tissues, such as the synovium and subchondral bone, is critical for the treatment of OA.

## Conclusions

This study demonstrates that knee loading at moderate intensity can suppress the activities of collagenases, gelatinases, and MMP13, in which Rac1-mediated p38 MAPK signaling is involved in a loading intensity-dependent fashion. The results suggest that moderate knee loading could be beneficial not only in enhancing bone formation and accelerating healing of bone fracture but also in ameliorating OA-linked tissue degradation.

## Abbreviations

ADAMTS: A disintegrin and metalloproteinase with thrombospondin motifs; BMC: Bone mineral content; BMD: Bone mineral density; CFP: Cyan fluorescent protein; FRET: Fluorescence resonance energy transfer; MAPK: Mitogen-activated protein kinase; MMP13: Matrix metalloproteinase 13; NFκB: Nuclear factor kappa B; OA: Osteoarthritis; YFP: Yellow fluorescent protein.

## Competing interests

The authors declare that they have no competing interests.

## Authors’ contributions

KH, PZ, HBS, and HY designed the study. KH, LZ, AC, TRD, QW, and HS collected the data. KH, PZ, SN, CCL, and HY interpreted the results. KH, JWS, and HY prepared the first version of the manuscript. All the authors reviewed the draft versions and gave their approval of the final version of the manuscript.

## Pre-publication history

The pre-publication history for this paper can be accessed here:

http://www.biomedcentral.com/1471-2474/14/312/prepub
